# Serum Troponin T Concentrations Are Frequently Elevated in Advanced Skin Cancer Patients Prior to Immune Checkpoint Inhibitor Therapy: Experience From a Single Tertiary Referral Center

**DOI:** 10.3389/fmed.2021.691618

**Published:** 2021-07-05

**Authors:** Jonas K. Kurzhals, Tobias Graf, Katharina Boch, Ulrike Grzyska, Alex Frydrychowicz, Detlef Zillikens, Patrick Terheyden, Ewan A. Langan

**Affiliations:** ^1^Department of Dermatology, University of Lübeck, Lübeck, Germany; ^2^Department of Cardiology, University of Lübeck, Lübeck, Germany; ^3^Department of Radiology and Nuclear Medicine, University of Lübeck, Lübeck, Germany; ^4^Dermatological Sciences, University of Manchester, Manchester, United Kingdom

**Keywords:** myocarditis, immune checkpoint inhibition, melanoma, non-melanoma skin cancer, troponin

## Abstract

Immune checkpoint inhibitor (ICI) therapy has revolutionized the treatment of several human malignancies, particularly metastatic skin cancer. However, immune-related myocarditis (irM), an immune-mediated adverse event (irAE), is often fatal. In the absence of a reliable biomarker, measurement of pre-ICI therapy serum troponin concentration has been proposed to identify patients at risk of developing irM, although real-world studies examining this strategy are lacking. Thus, we retrospectively analyzed the case records of all patients who commenced ICI therapy between January 2018 and December 2019 in a single university skin cancer center (*n* = 121) to (i) determine the incidence of irM, (ii) establish the frequency of pretreatment serum hsTnT elevations, and (iii) to establish whether this identified patients who subsequently developed irM. Only one patient developed irM, resulting in an overall incidence of 0.8%. Pretreatment hsTnT was measured in 47 patients and was elevated in 13 (28%). Elevated serum hsTnT concentrations were associated with chronic renal failure (*p* = 0.02) and diabetes (*p* < 0.0002). Pretreatment hsTnT was not elevated in the patient who developed fulminant irM. Pre-immunotherapy serum hsTnT concentrations were often asymptomatically elevated in patients with advanced skin cancer, none of whom subsequently developed irM during ICI therapy. However, large studies are required to assess the positive and negative predictive values of hsTnT for the development of irM. In the meantime, elevated hsTnT concentrations should be investigated before initiation of immunotherapy and closely monitored during early treatment cycles, where the risk of irM is greatest.

## Introduction

Immunotherapy, targeting specific immune checkpoints including cytotoxic T-lymphocyte antigen 4 (CTLA-4), programmed cell death 1 (PD1), and programmed cell death ligand 1 (PD-L1) has revolutionized the treatment of both locally advanced and metastatic melanoma and non-melanoma skin cancer (1, 2). As a result, there have been dramatic improvements in progression-free (PFS) and overall survival (OS), particularly in metastatic melanoma, with 5-years OS rates of over 50 and 44% for those treated with combined anti-CTLA4 and anti-PD1 therapy or anti-PD-1 monotherapy, respectively (3). Furthermore, the use of checkpoint inhibitors to prevent melanoma recurrence (4, 5), the adjuvant setting, is already leading to their increased use.

However, any decision to commence immune checkpoint inhibitor treatment must include a careful assessment of treatment-associated risks, particularly in the adjuvant setting where there is no radiological evidence of residual tumor. In fact, due to the lack of reliable biomarkers, it is not currently possible to predict which individual patients with fully resected high-risk metastatic melanoma will develop disease recurrence. Reassuringly, the safety profile of pembrolizumab is similar irrespective of whether it is administered in the adjuvant or palliative setting (5) and adjuvant nivolumab has a superior safety profile to ipilimumab in resected stage III and IV melanoma (4).

Immune-related adverse events (irAEs), side effects due to the removal of immune checkpoint inhibition, can essentially affect any tissue but commonly involve the gastrointestinal, endocrine, integumentary, hepatic, and respiratory systems with varying frequencies (6, 7). The majority of these irAEs can be managed with systemic corticosteroids. While cutaneous irAEs are common (8) and may even correlate with treatment response, (9) cardiac irAEs are rare. However, along with neurological irAEs, they account for almost 50 % of fatalities following ICI (10).

Cardiac irAEs often present early during treatment. There is also evidence that the incidence of immune-related myocarditis (irM), with mortality that can exceed 50%, is increasing (10–14). Although the incidence of irM is often cited as <1%, this may in fact be underestimated due to its non-specific initial presentation and rapidly fatal clinical course (15–18). Indeed, the endomyocardial biopsy, the gold standard for the diagnosis of immune-related myocarditis (irM), may also fail to confirm the diagnosis due to the patchy nature of the T-cell infiltrate, centered on areas of myocardial necrosis (19). Given the nonspecific clinical presentation of irM, combined with the lack of highly sensitive and specific diagnostic tests, a recent expert consensus statement emphasized the need for increased clinical awareness of irM (19).

Due to the lack of specific biomarkers for irM, there have been efforts to identify which patients may be most at risk. Lyon et al. (20) suggested that immune combination therapy (anti-PD1 plus anti-CTLA4), preexisting cardiac disease, previous autoimmune disease, and the expression of cardiac antigens in the tumor tissue may all predispose patients to immune checkpoint-mediated cardiotoxic effects (20). The extent to which preexisting cardiac disease predisposes to the development of irM remains unclear (2). For example, although this is often cited as a potential risk factor, a database analysis of over 100 patients with irM did not reveal widespread reporting of preexisting cardiac comorbidities (21). Several studies have reported that more than two-thirds of the cases of irM are in men, suggesting that the male sex may also be an important risk factor (16, 22).

The difficulty in identifying at-risk patients is confounded by the varied clinical presentation of irM, which may result in diagnostic delay. In fact, irM may be asymptomatic or present with symptoms ranging from non-specific fatigue and dyspnea to dysrhythmia and fulminant cardiogenic shock (23, 24). Interestingly, in contrast to several other irAEs, irM often presents shortly after treatment initiation, with the median time from the first infusion to initial symptoms being just over 1 month (25). Moslehi et al. reported that 64% of patients with irM developed the symptoms after the first or second dose of immune checkpoint therapy, although presentation after 33 treatment cycles has been reported (21, 22). Based on a pro-and retrospective register of patients who developed irM, Mahmood et al. found elevated high-sensitivity Troponin T (hsTnT) levels and an abnormal ECG in 94 and 89% of patients, respectively (16). Just over half of the patients had a normal left ventricular ejection fraction on echocardiography, and two-thirds of patients had raised serum n-terminal pro-brain natriuretic peptide (NT-proBNP) levels (16). While cardiovascular MRI using the Lake Louise Criteria has become the widely accepted clinical standard for diagnostic imaging of acute myocarditis (26, 27), its diagnostic performance in irM is still the subject of research and warrants larger studies (19). Given the limited specificity and sensitivity of electrocardiographic, echocardiographic, biochemical, radiological, and histological investigations in suspected irM, making the diagnosis of irM requires a high index of suspicion (19).

In the absence of an evidence-based surveillance strategy for the early detection of irM, routine measurement of serum Troponin concentrations prior to ICI therapy, and prior to cycles 2–4 in high-risk patients, has been proposed (20). Therefore, given that we introduced routine serum hsTnT testing prior to immune checkpoint therapy in 2019, we retrospectively analyzed all patients treated with immune checkpoint therapy between 2018 and 2019 to (i) investigate the incidence of irM, (ii) establish the frequency of pretreatment hsTnT elevations, and (iii) establish whether this identified patients who subsequently developed irM.

## Methods

In order to determine the incidence of irM in our center, we retrospectively analyzed the case notes of all patients in whom treatment with immunotherapy was initiated for locally advanced and/or metastatic melanoma, in both the adjuvant and palliative settings, and non-melanoma skin cancer in 2018 or 2019. All data were anonymized and collated and analyzed after approval by the ethics committee of the University of Luebeck and according to the Declaration of Helsinki principles (AZ 20-216). In addition to routine measurement of serum creatine kinase levels, we began routinely measuring serum hsTnT concentrations in all patients prior to immunotherapy in 2019. Serum NT-proBNP levels were determined depending on the existence of preexisting cardiac disease and when clinically indicated as part of the assessment of symptoms and signs which may have suggested heart failure. Data on sex and preexisting cardiac disease were collated given that these may be potential risk factors for the development of irM. In addition, age, cancer type, treatment type (anti-PD1, anti-PD-L1, combined anti-CTLA4/anti-PD1) and setting (adjuvant vs. palliative), and baseline electro- and/or echocardiography findings were also recorded. Overall survival (OS) was also calculated and compared between patients with normal vs. elevated serum hsTnT concentrations. Finally, any therapeutic consequences and the treatment of irM were noted. All statistical analyses were performed using Microsoft Excel (version 2019), and survival analyses were calculated using GraphPad Prism (version 8). *P* < 0.05 were considered statistically significant.

## Results

Between the 1st of January 2018 and the 31st of December 2019, a total of 121 patients received ICI therapy for locally advanced or metastatic melanoma and non-melanoma skin cancer (Flowchart). Eighty-one patients were male, and 40 patients were female, with a mean age of 74 years. The vast majority of the patients (96%) were treated for melanoma. Of these 116 patients, almost two-thirds were treated in the palliative setting for high-risk resected melanoma (stage IV), and the remaining third received ICI therapy in the adjuvant context (Table 1). Of the 77 patients receiving palliative treatment, 47 received combined anti-CTLA4 and anti-PD1 therapy, with the remaining patients receiving monotherapy with pembrolizumab (9) or nivolumab (21). Five patients with non-melanoma skin cancer were treated with immune checkpoint inhibitors, two with locally advanced squamous cell carcinoma (cemiplimab, anti-PD1), and three with metastatic Merkel cell carcinoma (avelumab, anti-PD-L1).

**Flow Chart:**
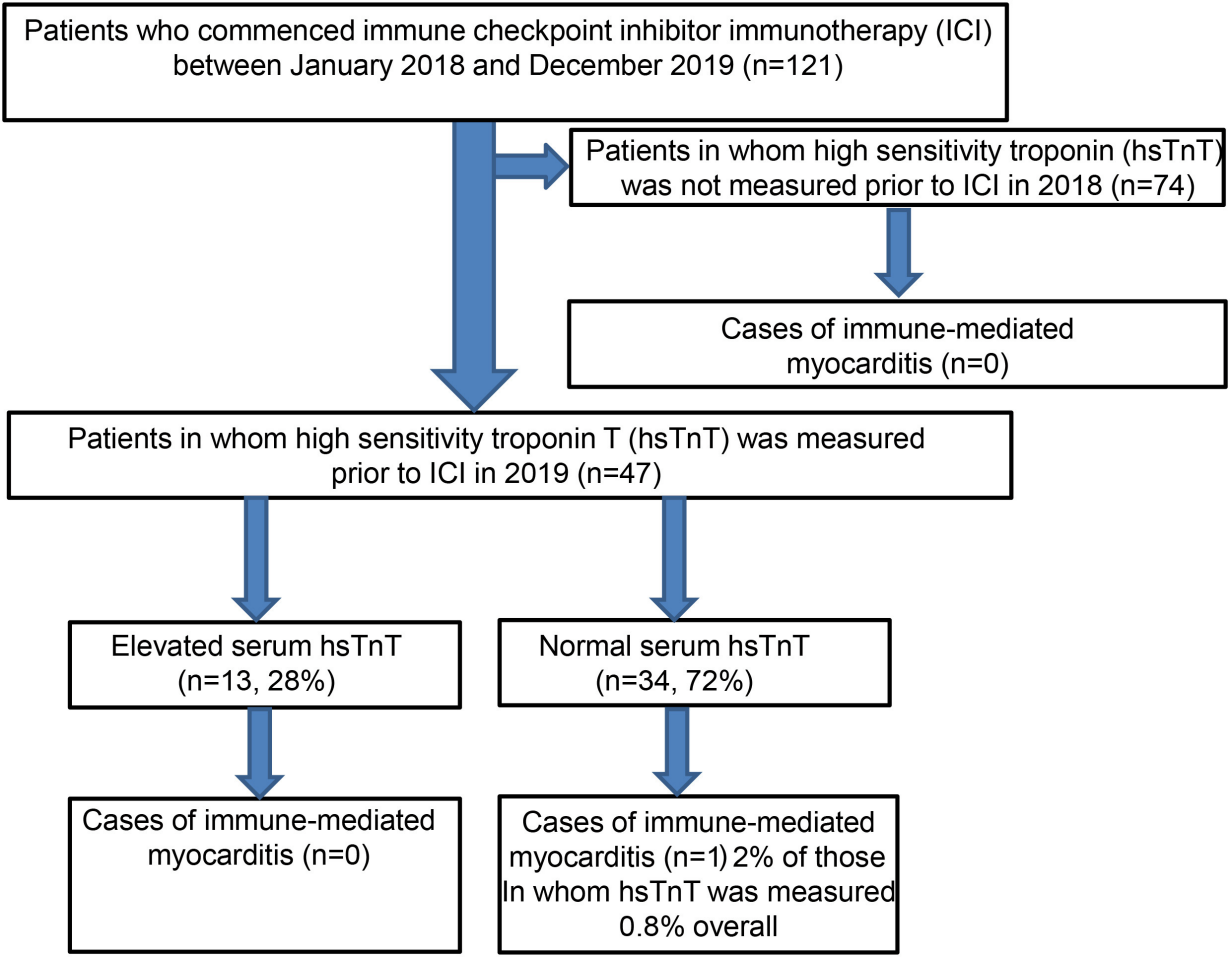
Study population.

**Table 1:** Distribution of sex, cancer type, and therapy setting of all patients.

**Sex**	**Males-80**	**Females-41**	
Cancer	Melanoma-116	Squamous cell carcinoma-2	Merkel cell carcinoma−3
Therapy setting	Palliative-79	Adjuvant-42	

**Figure 1:**
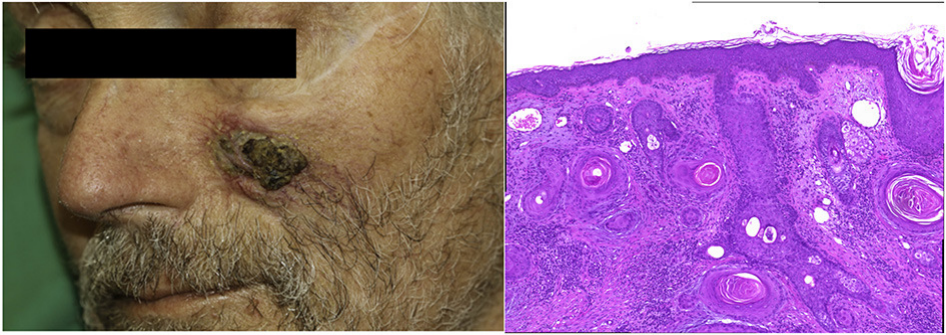
Clinical presentation and histopathology of squamous cell carcinoma. **(A)** 3 × 3 cm solitary subcutaneous hardened plaque with central ulceration. **(B)** Squamous cell carcinoma (H&E staining, 200×).

**Figure 2:**
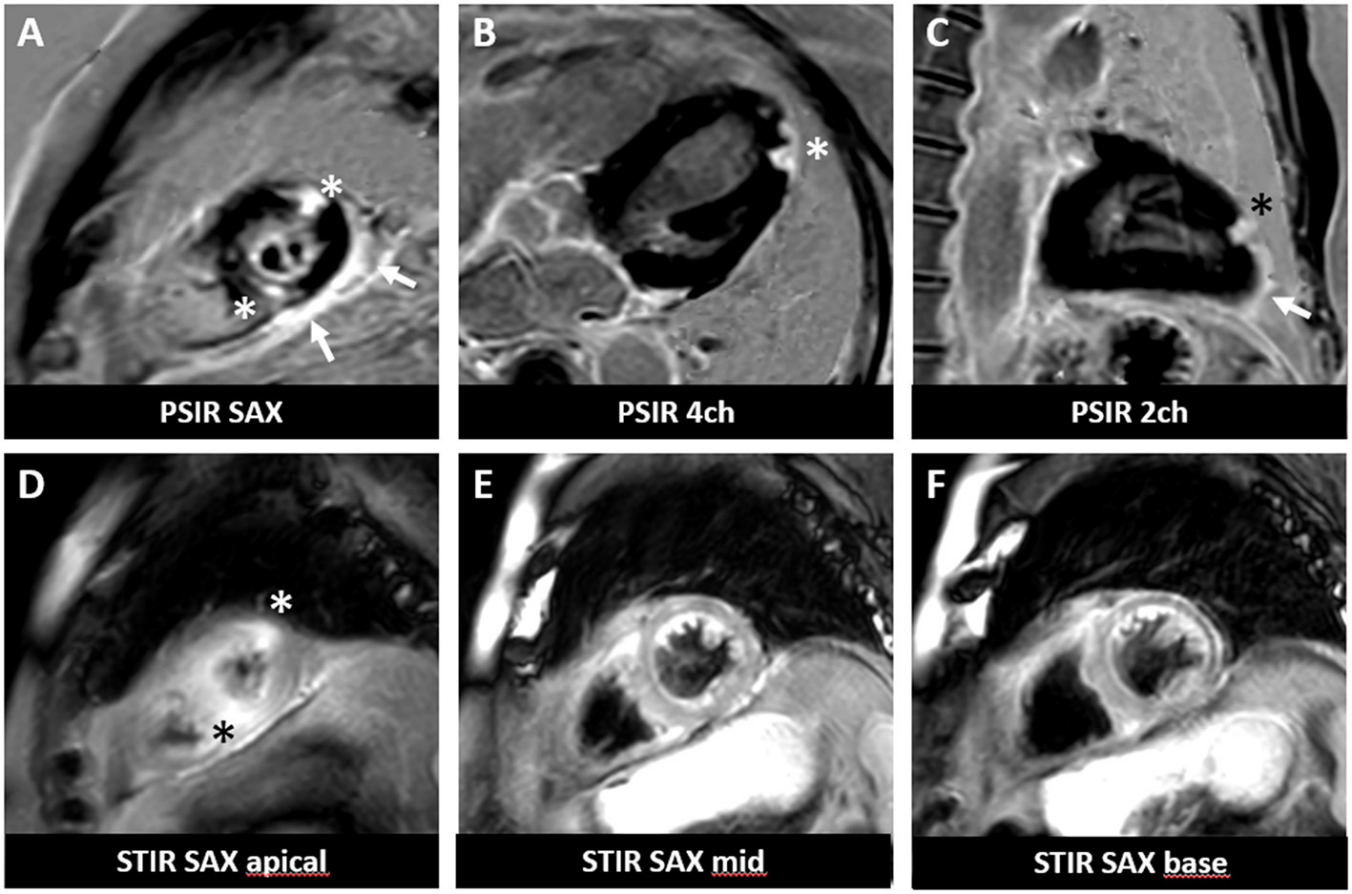
Cardiac magnetic resonance imaging of a patient with irM following a single infusion of cemiplimab. Cardiac MR revealed focal subepicardial to mid myocardial delayed gadolinium enhancement **(A–C)** associated with edema **(D–F)** at the lateral and inferoseptal apex (asterisks) involving the pericardium (arrows) in a delayed gadolinium enhancement sequence performed according to clinical standard. PSIR, phase-sensitive inversion recovery; STIR, short tau inversion recovery; SAX, short-axis view; 4ch, 4-chamber view; 2ch, 2-chamber view.

As expected, the overall incidence of irM was low (0.8%) and in line with that reported in the published literature (16, 20). Only a single patient developed irM which developed 16 days after the first dose (350 mg i.v.) of cemiplimab for locally advanced squamous cell carcinoma (Figure 1). The patient presented with generalized myalgia and malaise. Admission ECG was unremarkable, but the serum hsTnT concentration was markedly elevated at 457 ng/l. Creatine kinase and NT-proBNP were also elevated at 4596 U/L and 901 pg/ml, respectively. His peak hsTnT and NT-proBNP levels reached 2238 ng/l (normal limit <14) and 1366 pg/ml (normal limit <486) at 32 and 44 days, respectively, after the first dose of cemiplimab. An echocardiogram revealed left ventricular dysfunction. The patient was admitted to the coronary care unit for monitoring and high-dose immunosuppression with intravenous prednisolone (2 mg/kg). Although the patient declined an endomyocardial biopsy, cardiac MR imaging demonstrated a focal transmural, almost global subendocardial myocardial edema, and an epi- to mid-myocardial enhancement with pericardial involvement, consistent with irM (Figure 2). Despite an initial improvement, the patient's condition deteriorated and additional immunosuppression with mycophenolate mofetil (3 g/d) was commenced. The patient's recovery was complicated by *Staphylococcus aureus* sepsis and reactivation of cytomegalovirus infection. Following antibiotic and antiviral treatment, along with tapering of his immunosuppressive therapy, the patient was discharged to a rehabilitation unit after 68 days of in-patient care. Following 4 weeks of rehabilitation, the patient was discharged home but died 4 weeks later of cardiac failure, some 20 weeks after the administration of cemiplimab.

**Figure 3:**
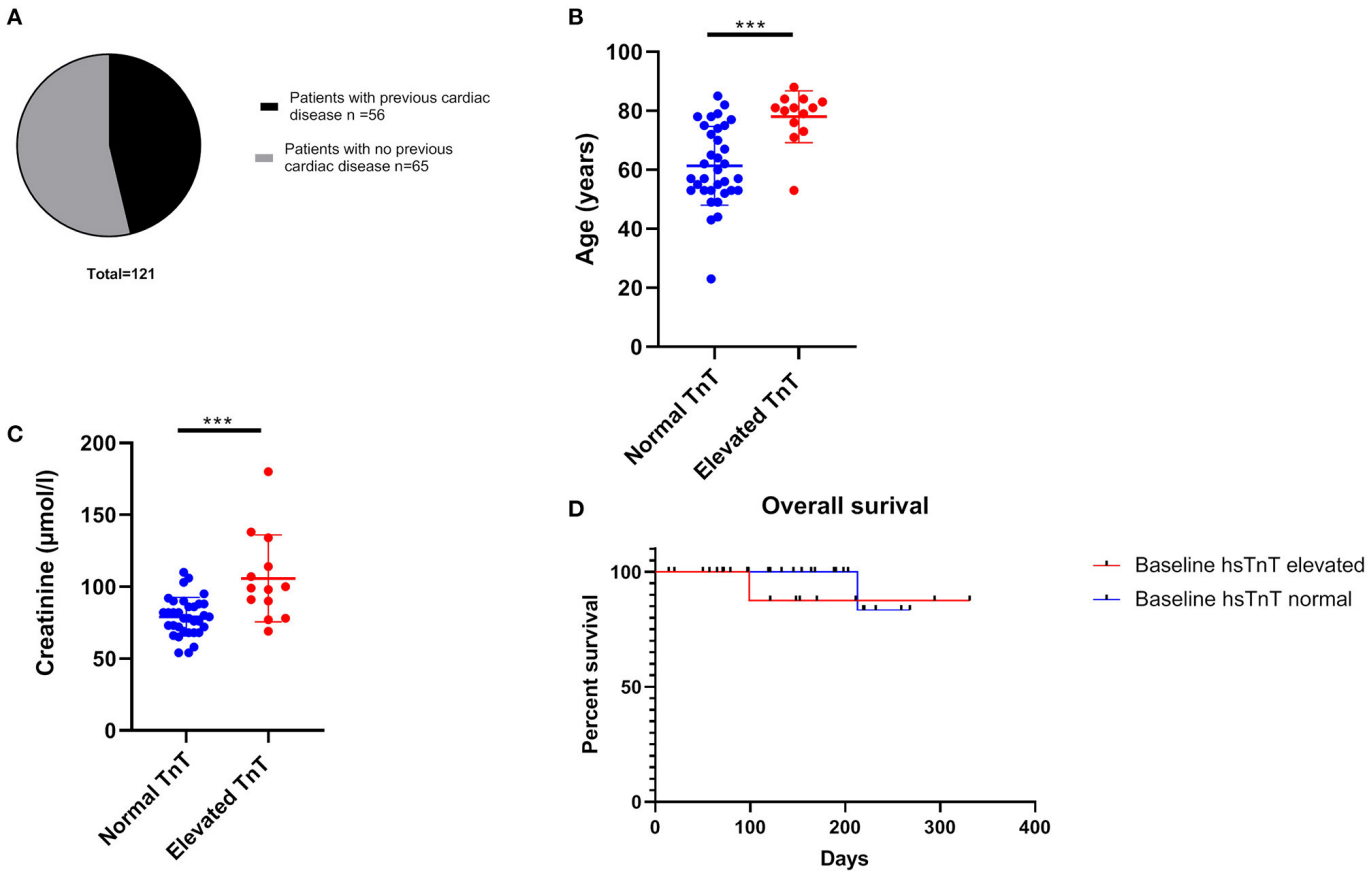
Cardiac co-morbidity status and factors associated with elevated hsTnT concentrations. **(A)** Almost 50% of all patients had pre-existing ischaemic heart disease. Age (**B**) and elevated baseline creatinine concentration **(C)** were significantly associated with increased hsTnT levels ***p < 0.001. **(D)** overall survival was not significantly different between the elevated and normal hsTnT groups.

Fifty-six out of 121 patients had preexisting cardiac comorbidities before initiating immunotherapy (Figure 3A). Baseline echocardiography was available for 59 patients, which were abnormal in 33 patients. Given that we introduced routine pre-immunotherapy baseline hsTnT measurement in 2019, based on the American Society of Clinical Oncology (ASCO) guidelines (28), we were able to collect data for 47 patients (Table 2). HsTnT was measured using the Elecsys Assay (Roche), according to the manufacturer's instructions, and was elevated in 28% of patients (13 out of 47) in the absence of any clinical symptoms. Ten had preexisting cardiac comorbidities (77%), including arrhythmias, chronic heart failure, and coronary artery disease. Five of those patients had additionally elevated baseline creatinine levels (38%), and 46% had elevated NT-proBNP natriuretic-peptide concentrations.

**Table 2:** Demographics and factors associated with normal and elevated baseline hsTnT concentrations.

**Patients' baseline characteristiscs**	**Troponin elevated**	**Troponin normal**
**Sex**		
Male	9	22
Female	4	12
**Age**		
Mean	78	61.4
Range	53–88	23–85
**Tumor type**		
Melanoma	13	33
Squamous cell carcinoma	0	1
**Baseline hsTnT**		
Mean (ng/l)	25.5	7.04
Range (ng/l)	14–50.7	5–13.4
**Baseline creatinine**		
Mean (μmol/l)	105.8	79.1
Range (μmol/l)	69–180	54–110
**Echocardiography**		
Abnormal echocardiography	7	3
Normal echocardiography	2	13
Not performed	4	18
**Previous cardiac disease**		
	10 out of 13	8 out of 34
**Immunotherapy**		
First combinated therapy, afterwards PD-1 Inhibitor	6	13
Nivolumab monotherapy	6	7
Pembrolizumab monotherapy	1	13
Cemiplimab	0	1
**BRAF mutation**		
BRAF positive	2	13
BRAF negative	11	21
**Diabetes mellitus**		
Co-existing Diabetes mellitus Type 2	4	1
No history of Diabetes mellitus Type 2	9	33
**Therapeutic setting**		
Adjuvant	4	18
Palliative	9	16
**Immunotherapy related myocarditis (irM)**		
Events	0	1

Each patient with elevated serum hsTnT concentration was assessed, often on an emergency basis, and serial hsTnT measurements, ECGs, and echocardiography were performed to exclude acute ischemia (Table 3). There was no evidence of acute ischemia in any of the patients, and immunotherapy was subsequently initiated as planned after cardiological evaluation. Reassuringly, none of the patients with elevated pretreatment hsTnT concentrations developed any signs of cardiotoxicity in general or myocarditis in particular during ICI therapy.

**Table 3:** Cardiological assessment in patients with elevated hsTnT concentrations.

**Patient**	**Initial hsTnT (ng/l)**	**Follow-up hsTnT (ng/l)**	**NT-proBNP (ng/l)**	**ECG**	**Echocardiography performed**	**Creatinine (μmol/l)**	**Cardiological Evaluation**
	**Normal range <14 ng/l**	**Normal range <14 ng/l**	**Normal range <486 ng/l**			**Normal range 59–104 μmol/l**	
1	37.2	36	-	No evidence of acute ischaemia	Yes	138	hsTnT elevation due to chronic renal impairment
2	15	13.2	1,216	No evidence of acute ischaemia	Yes	98	hsTnT due to pre-existing cardiac disease
3	25.9	18.9	1,654	No evidence of acute ischaemia	Yes	134	hsTnT due to pre-existing cardiac disease/chronic renal impairment
4	25	23.2	-	No evidence of acute ischaemia	No	77	No evidence of ischaemic heart disease
5	34.8	34.0	4,081	No evidence of acute ischaemia	Yes	100	hsTnT due to pre-existing chronic cardiac failure
6	32.5	30.5	1,395	No evidence of acute ischaemia	Yes	69	No evidence of ischaemic heart disease
7	28.4	28.8	-	No evidence of acute ischaemia	Yes	78	No evidence of ischaemic heart disease
8	18.2	14.0	-	No evidence of acute ischaemia	No	180	hsTnT elevation due to chronic renal impairment
9	15.2	14.5	-	No evidence of acute ischaemia	No	112	hsTnT elevation due to chronic renal impairment
10	50.7	45.8	700	No evidence of acute ischaemia	Yes	91	No evidence of ischaemic heart disease
11	21.9	17.6	-	No evidence of acute ischaemia	No	120	hsTnT elevation due to chronic renal impairment
12	20.9	18.9	1,800	No evidence of acute ischaemia	Yes	95	hsTnT due to pre-existing chronic cardiac failure
13	14.0	-	-	No evidence of acute ischaemia	Yes	107	hsTnT due to pre-existing cardiac disease

Of the 34 patients with normal pretreatment hsTnT concentrations, one patient developed myocarditis. Preexisting diabetes and ischemic heart disease were significantly associated with an elevated serum hsTnT level concentration (*p* = 0.02 and *p* < 0.0002, respectively). There was no association between hsTnT concentration and sex or BRAF status (in patients with melanoma) (Fisher's exact test). Patients with elevated hsTnT levels were significantly older (Figure 3B) and had significantly increased serum creatinine levels (Figure 3C). HsTnT did not affect OS, although changes in cancer survival were not expected due to the relatively short follow-up period (Figure 3D).

## Discussion

Immunotherapy can produce significant and durable antitumor responses in a range of locally advanced and metastatic skin cancers (29). The increasing use of immunotherapy is likely to result in clinicians from several specialties being confronted with potentially fatal irAE (30). The life-threatening nature of irM, compounded by the difficultly of prompt recognition to initiate rapid treatment, makes it one of the most challenging irAEs to manage successfully (16). Given that myocarditis frequently occurs shortly after initiation of immunotherapy, it is possible that certain patient groups are more susceptible, potentially related to preexisting cardiac risk factors (31).

Evidence from animal models suggests that both CTLA-4 and PD-1 may have protective effects against stress (32–34) and that PD-1 ligands can protect the myocardium. For example, CTLA-4 knockout mice reportedly develop autoimmune myocarditis caused by CD8+T cells, whereas knockout of PD-1 is associated with anti-cTn autoantibody-mediated myocarditis (31). The extent to which pharmacological manipulation of the PD-1/PD-L1 pathway influences the treatment of immune-mediated cardiac inflammation is unclear (31, 35). Consistent with the published literature, irM was rare in our cohort.

Several recent publications have recommended close hsTnT surveillance before the initiation of ICI therapy and during the early treatment cycles, particularly when combined anti-CTLA4 and anti-PD1 therapy is planned (28, 36, 37). In our cohort, this recommendation identified asymptomatic elevations of serum hsTnT levels in 28% of patients with advanced skin cancer. In the single case of irM which occurred, baseline biochemical (including serum hsTnT levels), ECG, and echocardiographic findings were unremarkable. The only identifiable potential risk factor in this patient was being male (2, 22). Importantly, the patient had no history of renal impairment, cardiac disease, or diabetes. Recent studies, both cross-sectional and longitudinal, have highlighted the association between elevated hsTnT levels and diabetes mellitus, consistent with our findings (38, 39). HsTnT has even been proposed as a predictor of incident diabetes (40). The mechanism underlying this association is currently unclear but may be mediated by concurrent chronic renal impairment or reflect microangiopathy-associated structural nerve damage in type II diabetes mellitus (41, 42).

Elevated cardiac biomarkers, including hsTnT, have been reported in cancer patients prior to anticancer therapy and are strongly related to all-cause mortality. However, the studies to date have largely included patients with breast, lung, and hematological malignancies (43–45). In one study of over 550 cancer patients, only two (0.4%) patients with advanced skin cancer were included (45). It is therefore of note that over a quarter of the patients in our study had asymptomatic elevated hsTnT levels, the vast majority of whom had metastatic melanoma. The extent to which metastatic melanoma *per se* is associated with increased serum hsTnT concentrations needs to be confirmed in larger studies. Although there was no difference in overall survival between the group with normal and that with elevated hsTnT concentrations, the observation period was too short to allow any conclusions to be drawn on whether patients with elevated pretreatment hsTnT concentrations had a poorer overall prognosis, potentially independent of the development of irM.

In this context, it is particularly interesting to note that the association between irAEs and response to treatment with ICI may be compounded by both a publication and an immortal time bias. Therefore, prospective studies in the adjuvant treatment setting may be best placed to conclusively determine the relationship between toxicity and response in patients undergoing ICI (46). Future multicenter studies should examine the extent to which elevated serum hsTnT concentrations may identify “at-risk” patients not only for irM but also for all-cause mortality. Our study was neither designed nor powered to evaluate the positive or negative predictive value of pretreatment elevated serum hsTnT for the development of irM, which would also require large multicenter studies. Additional limitations of our study include the predominance of a single cancer type (melanoma), the single-center setting, its retrospective nature, and that only one patient developed irM. Moreover, as we only measured pretreatment serum hsTnT concentrations our study does not allow any conclusions to be drawn about the sensitivity or specificity of serum hsTnT concentrations in the diagnosis of irM. Finally, the patient declined an endomyocardial biopsy, which may have shed light on the extent and nature of the immune-cell infiltrate.

Nevertheless, in our experience, pre-therapeutic elevated hsTnT concentrations were not associated with the development of irM. This may provide some reassurance to treating physicians and patients. Given the dramatic increase in the number of cancer patients who are now eligible for treatment with immune checkpoint inhibitors (47), there will inevitably be more patients who develop irAEs. A key challenge over the next decade will be the identification of biomarkers not only to maximize the benefit of immunotherapy among patients receiving it but also to maximize patient safety and optimize treatment of irAEs, especially those associated with significant morbidity and mortality. To this end, interleukin 6, C-reactive protein, and melanoma inhibitory activities have recently been reported to correlate with the onset of irAEs (48). Should these results be confirmed, a role for anti-IL-6R antibodies in the treatment of irAEs may emerge.

In summary, we confirm that irM is rare and report that pre-ICI treatment hsTnT concentrations were frequently elevated in patients with advanced skin cancer in the absence of acute ischemia. Following cardiac evaluation, immunotherapy was administered as planned and none of the patients with an elevated hsTnT concentration developed irM, although this was not expected given the sample size. Nevertheless, pre-ICI treatment hsTnT concentrations should be routinely performed before the initiation of immune checkpoint inhibition (28, 36, 37) and thoroughly investigated when elevated. Following cardiological assessment and the decision to initiate ICI therapy, pre- and early-treatment serum hsTnT concentrations should be measured and closely monitored especially during the initial treatment cycles where the risk of irM is greatest, particularly in patients with additional risk factors for irM, including the male sex, diabetes, a history of heart disease, and those undergoing combined anti-PD1 and anti-CTLA4 immunotherapy.

## Data Availability Statement

The original contributions presented in the study are included in the article. Further inquiries can be directed to the corresponding author.

## Ethics Statement

The studies involving human participants were reviewed and approved by University of Lübeck, Ref 20-216. Written informed consent for participation was not required for this study in accordance with the national legislation and the institutional requirements. Written informed consent was obtained from the individual(s) for the publication of any potentially identifiable images or data included in this article.

## Author Contributions

JK, EL, and PT conceptualized the study and analyzed the data. JK recorded the data. UG and AF reported the MRI result and provided the images. KB evaluated the histology and provided the images. EL and JK wrote the manuscript. All co-authors reviewed and revised it.

## Conflict of Interest

EL reports personal fees and non-financial support from Bristol Myers Squibb, personal fees and non-financial support from Novartis, Meeting and travel support from Curevac, and advisory board fees from Sun Pharma. PT speaker's honoraria from BMS, Novartis, MSD, Pierre-Fabre, CureVac, and Roche, consultant's honoraria from BMS, Novartis, Pierre-Fabre, Merck Serono, Sanofi, and Roche and travel support from BMS, Pierre-Fabre, and Roche. The remaining authors declare that the research was conducted in the absence of any commercial or financial relationships that could be construed as a potential conflict of interest.
